# Generation of transgenic cynomolgus monkeys that express green fluorescent protein throughout the whole body

**DOI:** 10.1038/srep24868

**Published:** 2016-04-25

**Authors:** Yasunari Seita, Tomoyuki Tsukiyama, Chizuru Iwatani, Hideaki Tsuchiya, Jun Matsushita, Takuya Azami, Junko Okahara, Shinichiro Nakamura, Yoshitaka Hayashi, Seiji Hitoshi, Yasushi Itoh, Takeshi Imamura, Masaki Nishimura, Ikuo Tooyama, Hiroyuki Miyoshi, Mitinori Saitou, Kazumasa Ogasawara, Erika Sasaki, Masatsugu Ema

**Affiliations:** 1 Department of Stem Cells and Human Disease Models, Research Center for Animal Life Science, Shiga University of Medical Science, Seta, Tsukinowa-cho, Otsu, Shiga 520-2192, Japan; 2 JST, ERATO, Yoshida-Konoe-cho, Sakyo-ku, Kyoto 606-8501, Japan; 3 Department of Anatomy and Embryology, Faculty of Medicine, University of Tsukuba, Tennodai 1-1-1, Tsukuba, Ibaraki 305-8577, Japan; 4 Central Institute for Experimental Animals, 1430 Nogawa, Miyamae-ku, Kawasaki, Kanagawa 216-0001, Japan; 5 Department of Physiology, Shiga University of Medical Science, Otsu, Shiga 520-2192, Japan; 6 Department of Pathology, Shiga University of Medical Science, Otsu, Shiga 520-2192, Japan; 7 Department of Pharmacology, Shiga University of Medical Science, Otsu, Shiga 520-2192, Japan; 8 Molecular Neuroscience Research Center, Shiga University of Medical Science, Otsu, Japan; 9 Department of Physiology, School of Medicine, Keio University, 35 Shinanomachi, Shinjuku-ku, Tokyo 160-8582, Japan; 10 Department of Anatomy and Cell Biology, Graduate School of Medicine, Kyoto University, Yoshida-Konoe-cho, Sakyo-ku, Kyoto 606-8501, Japan; 11 Department of Reprogramming Science, Center for iPS Cell Research and Application, Kyoto University, 53 Kawahara-cho, Shogoin Yoshida, Sakyo-ku, Kyoto 606-8507, Japan; 12 Institute for Integrated Cell-Material Sciences, Kyoto University, Yoshida-Ushinomiya-cho, Sakyo-ku, Kyoto 606-8501, Japan; 13 PRESTO, Japan Science and Technology Agency (JST), 4-1-8 Honcho Kawaguchi, Saitama 332-0012, Japan

## Abstract

Nonhuman primates are valuable for human disease modelling, because rodents poorly recapitulate some human diseases such as Parkinson’s disease and Alzheimer’s disease amongst others. Here, we report for the first time, the generation of green fluorescent protein (GFP) transgenic cynomolgus monkeys by lentivirus infection. Our data show that the use of a human cytomegalovirus immediate-early enhancer and chicken beta actin promoter (CAG) directed the ubiquitous expression of the transgene in cynomolgus monkeys. We also found that injection into mature oocytes before fertilization achieved homogenous expression of GFP in each tissue, including the amnion, and fibroblasts, whereas injection into fertilized oocytes generated a transgenic cynomolgus monkey with mosaic GFP expression. Thus, the injection timing was important to create transgenic cynomolgus monkeys that expressed GFP homogenously in each of the various tissues. The strategy established in this work will be useful for the generation of transgenic cynomolgus monkeys for transplantation studies as well as biomedical research.

To date, many transgenic animals have been developed including mice^1^,^2^, rats^3^,^4^ and domestic animals^5^. Although many human disease models have used these transgenic animals, most used mice because the technique of genetic manipulation, such as generation of transgenic animals by DNA microinjection into pronuclear embryos^2^,^6^,^7^ and generation of gene-targeting animals by using homologous recombination in embryonic stem cells^8^,^9^, have been established. While rodents are valuable models for biomedical research, the evolutionary distance between rodents and humans is almost 87 million years^10^, and rodents do not always recapitulate human behavioural and biological responses^11^. For example, it is difficult to use mice as models for AIDS^10^, because the host range for human immunodeficiency virus is highly restricted; for influenza^11^ because the pathogenesis in mice is different from that in humans and influenza virus causes hypothermia^12^ and severe viral pneumonia^13^,^14^; and for lung disorders^15^ such as asthma, because the original findings of asthma obtained from mouse models had limited success in humans studies^16^. Additionally, the pathological and behavioural phenotypes of mouse genetic models are often quite different from the human condition. The inactivation of Parkinson’s disease (PD) genes *Pink1*^17^, *Parkin*^18^, *Dj-1*^19^, *Lrrk2*^20^ was insufficient to cause PD in mice. Triple knockout mice lacking *Parkin*, *DJ-1*, and *Pink1* have normal morphology, and normal numbers of dopaminergic and noradrenergic neurons in the substantia nigra^21^. Mouse knockout models of human tumour suppressor genes also often display a tumour spectrum at variance with the human pathology. For example, in humans, germline or somatic *RB* gene loss is associated with the development of retinoblastomas and osteosarcomas and, later in life, with small cell lung carcinomas, whereas mice with an *Rb* deletion fail to develop these types of tumours^22^. Accordingly, it is required to establish transgenic animal models to recapitulate human diseases.

Nonhuman primates (NHPs) are considered one of the most valuable animal models. Several NHPs are used as laboratory animals, including New World monkeys such as common marmosets, and Old World monkeys such as rhesus monkeys and cynomolgus monkeys. Common marmosets clearly exhibit anthropoid primate characteristics, are relatively inexpensive to maintain, mature by 1.5–2 years of age, produce next generation offspring by 3 years of age and give birth to 3–5 offspring per year^23^. However, marmosets exhibit physiological and functional differences relative to humans to a greater degree than do Old World primates. These differences include pituitary gonadotropin secretion and action^24^, their ability to maintain bone mass without the need for gonadal estrogen^25^, lack of age-related ovulation decline^26^ and high fasting glucose and triglyceride levels^27^. In contrast, Old World monkeys are closer to humans in organ size and structure and therefore have been used for disease models such as stroke^28^,^29^, Parkinson’s disease^30^,^31^, Huntington’s disease^32^,^33^ and transplantation studies^34^,^35^. Especially, cynomolgus monkeys are considered a useful animal model because they can be bred throughout the year in contrast to rhesus macaques that have seasonal breeding pattern.

Green fluorescent protein (GFP) is frequently used in biomedical research and GFP-expressing animals are an important source of bone marrow, spermatogonial stem cells and organ transplantation, and pre-implantation embryos used to produce chimeric embryos. After the first transgenic NHPs were created by the transduction of GFP retroviral vector in 2001^36^, a transgenic NHP model of Huntington’s disease was developed^37^. In 2009, the first transgenic NHPs with germline transmission were reported^38^. Recently, genome editing in monkeys using the CRISPR/Cas9 system^39^ and TALEN system^40^ was developed and used to generate human disease models^41^. Currently, GFP mice^42^, rats^43^, rabbits^44^,^45^, cats^46^, pig^47^, cattle^48^, common marmosets^38^ and rhesus monkeys^36^,^37^,^49^ have been produced, yet no GFP cynomolgus monkey has been generated.

We report here for the first time, the generation of a GFP-expressing cynomolgus monkey. Our data show that the use of a human cytomegalovirus immediate-early enhancer and chicken beta actin promoter (CAG) directed the ubiquitous expression of the transgene in cynomolgus monkeys. Furthermore, we show that lentiviral injection into mature oocytes before fertilization is an efficient way to create transgenic NHPs with homogenous expression of GFP in each tissue, including the amnion, and fibroblasts. We also clearly demonstrate the fluorescence in the transgenic foetus was due to GFP expression and not autofluorescence, by using wild type tissues as negative controls. This system will contribute to creating human disease models and is also a good tool for transplantation studies.

## Result

### Production of GFP transgenic cynomolgus monkeys

In rodents, the pronuclear injection of DNA into fertilized eggs is an efficient strategy to generate transgenic animals. An alternative method is the use of genetically engineered embryonic stem cells. However, these strategies have been unsuccessful in NHPs to date. Since a transgenic marmoset with the capability of germ line transmission has been generated by injecting lentivirus into the perivitelline space of a two-cell-stage embryo^38^, we followed the technique to create a transgenic cynomolgus monkey.

**Figure 1:**
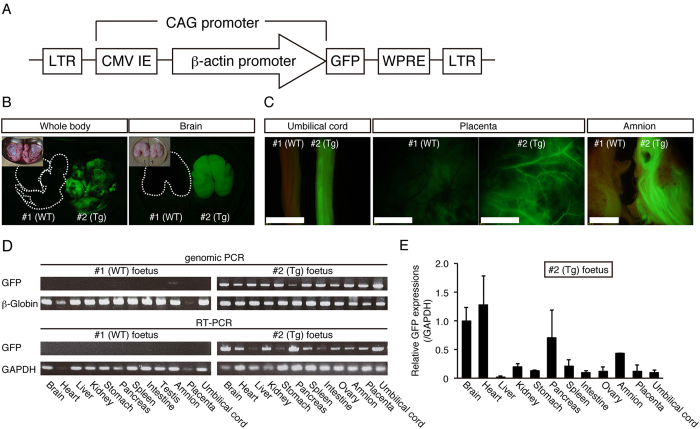
GFP expression of day 92 aborted twin foetus. (**A**) Schematic representation of the lentiviral vector used for the transduction of GFP. LTR: long terminal repeat. CMV IE: human cytomegalovirus immediate early enhancer. WPRE: woodchuck hepatitis posttranscriptional regulatory element. (**B**) Epifluorescence images of the whole body and brain in day 92 aborted twin foetuses (#1 and #2). Inset in each panel shows bright images. (**C**) Epifluorescence in umbilical cords, placenta and amnion in day 92 aborted twin (#1 and #2) foetuses. Each organ on the left was obtained from #1 foetus, whereas those on the right were from #2 foetus. Strong epifluorescence was observed under excitation light (489 nm). (Scale bar: 500 μm) (**D**). Genomic PCR (top panel) and RT-PCR (bottom panel) from #1 and #2 foetus organs. (**E**) GFP expression in #2 foetus organs evaluated by RT-qPCR. Data are represented as mean ± SD.

**Figure 2:**
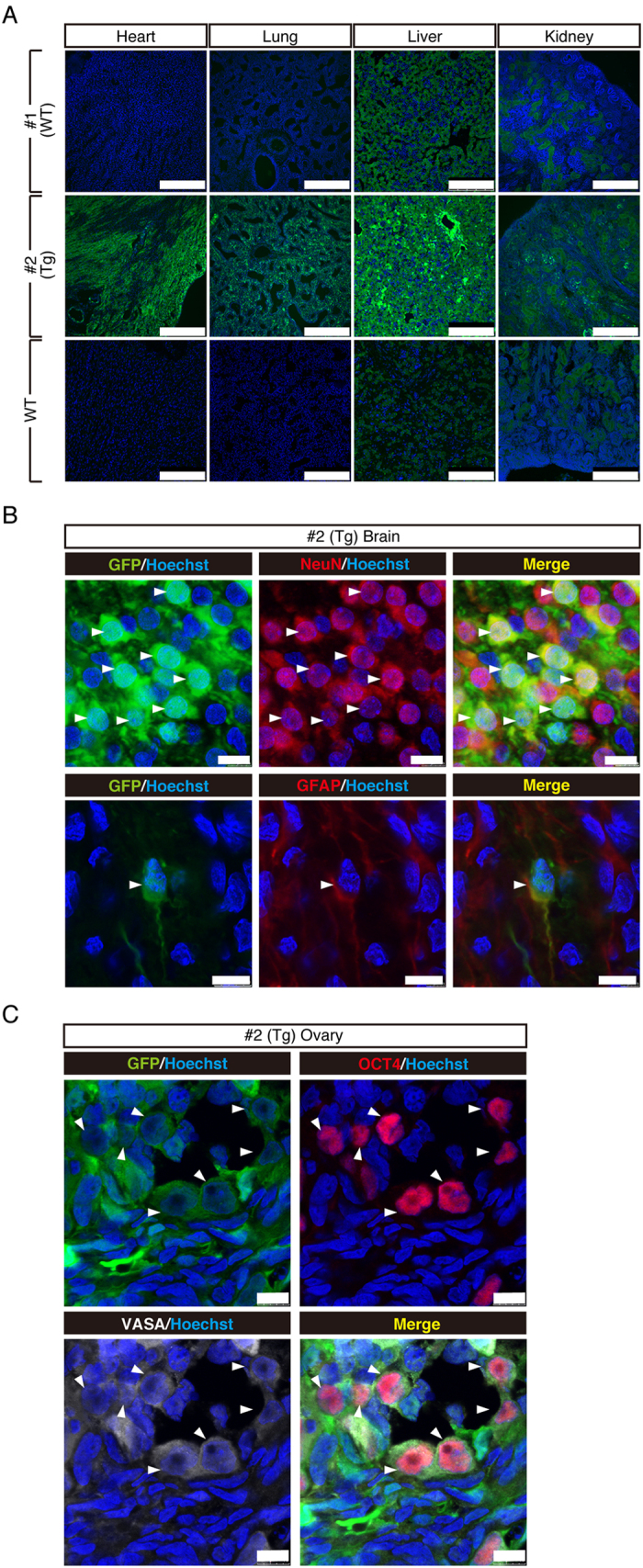
GFP protein expression in various tissues. (**A**) Immunohistochemistry of tissues from a day 92 aborted twin foetus detected by confocal microscopy. Note that #2 foetus shows overall strong GFP fluorescence in all the tissues examined, and that the WT liver and kidney showed moderate fluorescence (autofluorescence). Images were taken under the same instrumental settings (same laser intensity, etc). (Scale bar: 250 μm) (**B**). Immunohistochemical analysis of #2 brain with anti-GFP, anti-GFAP and anti-NeuN antibodies. Colocalisation of GFP and NeuN or GFAP are shown by white arrows. (Scale bar: 10 μm) (**C**). Immunohistochemical staining of #2 ovary with anti-OCT4 and VASA antibodies. Colocalisation of GFP and OCT4 or VASA are shown by white arrows. (Scale bar: 10 μm).

Firstly, we constructed a lentiviral vector that carries GFP cDNA under the control of human cytomegalovirus early enhancer and chicken beta actin (CAG) promoter (Fig. 1A), because CAG promoter is able to achieve ubiquitous expressions in various species^50^. Transfer of five lentivirus-injected embryos into three recipients resulted in two pregnancies, one of which ended in a miscarriage of twins on day 92 of gestation (Table 1). Detailed organ examination and histology identified no abnormalities (data not shown). Although one of the twins (#1) showed no detectable fluorescence, the other (#2) showed strong fluorescence in the face, skin, placenta and brain (Fig. 1B, data not shown). Strong fluorescence was observed in the organs of #2 foetus including the umbilical cord, placenta and amnion compared with #1 foetus under a fluorescent stereomicroscope (Fig. 1C). PCR analysis was performed to detect GFP transgene and demonstrated that the GFP transgene was integrated ubiquitously in the genome of all tissues in #2 foetus, but not #1 foetus. Consistent with this, RT-PCR analysis indicated that GFP mRNA was expressed ubiquitously in #2 foetus, but not #1 foetus (Fig. 1D). From these results, the #1 and #2 foetuses were determined to be wild type (WT) and transgenic (Tg), respectively. Abundant GFP mRNA expression was detected in the brain, heart and pancreas in #2 foetus, while moderate GFP expression was detected in the kidney, spleen and amnion by RT-quantitative PCR (RT-qPCR) (Fig. 1E). We confirmed GFP protein expression in these organs by immunohistochemistry with an anti-GFP antibody and found that fluorescence was detected in all these organs from the #2 foetus (Fig. 2A). Strong fluorescence was observed in the heart, lung, spleen and stomach from #2 foetus, but not #1 foetus or the miscarried WT foetus (Fig. 2A, Supplementary Fig. 1A), under the same instrumental settings (same laser intensity, please see Materials and Methods). However, we detected moderate fluorescence in the liver and kidney in #1 foetus that was WT as indicated by PCR analysis (Fig. 2A). To investigate carefully the nature of the fluorescence, we examined tissues from the liver and kidney of another miscarried WT foetus and found that these organs also had comparable fluorescence (Fig. 2A). Careful comparison revealed significant differences in the intensity and pattern of fluorescence between WT and #2 foetus, demonstrating that the fluorescence observed in the tissues of #1 and WT foetuses were autofluorescence and that the fluorescence in the #2 foetus was derived from GFP protein. Regarding autofluorescence, as laser power increased, autofluorescence of hepatocytes in the liver and renal tubules in the kidney also increased (Supplementary Fig. 1B). WT monkey face exhibited moderate autofluorescence as if it is GFP transgenic monkey (Supplementary Fig. 1C), showing the importance of negative control. Taken together, we concluded that it was very important to compare fluorescence of WT and Tg at the same time, and #2 foetus was Tg with ubiquitous overexpression of the GFP protein.

### GFP expression in the brain tissue

To investigate the detailed expression of GFP in the brain of a day 92 foetus, we examined the colocalisation of GFP, a radial glial and astroglial marker, glial fibrillary acidic protein (GFAP), and a neuronal specific nuclear protein marker, NeuN (Fig. 2B). Widespread GFP expression was observed throughout the cortex, including the forebrain, midbrain, and hindbrain. At a higher magnification, neuronal GFP expression was identified by its colocalisation with NeuN in the prefrontal cortex (Fig. 2B). GFP and GFAP were also colocalised in the lateral ventricle (Fig. 2B).

### Germline transmission of the transgene

To investigate whether the transgene was transmitted to the germline, we examined the colocalisation of GFP and VASA (DDX4), a marker for primordial germ cell (PGC) of the ovary in the day 92 foetus. GFP and VASA were clearly colocalised in a subset of the ovary cells (Fig. 2C). Expression of OCT4, another marker for PGC was also well overlapped with that of GFP (Fig. 2C).

### Partial GFP expression in the peripheral blood and fibroblasts

**Figure 3:**
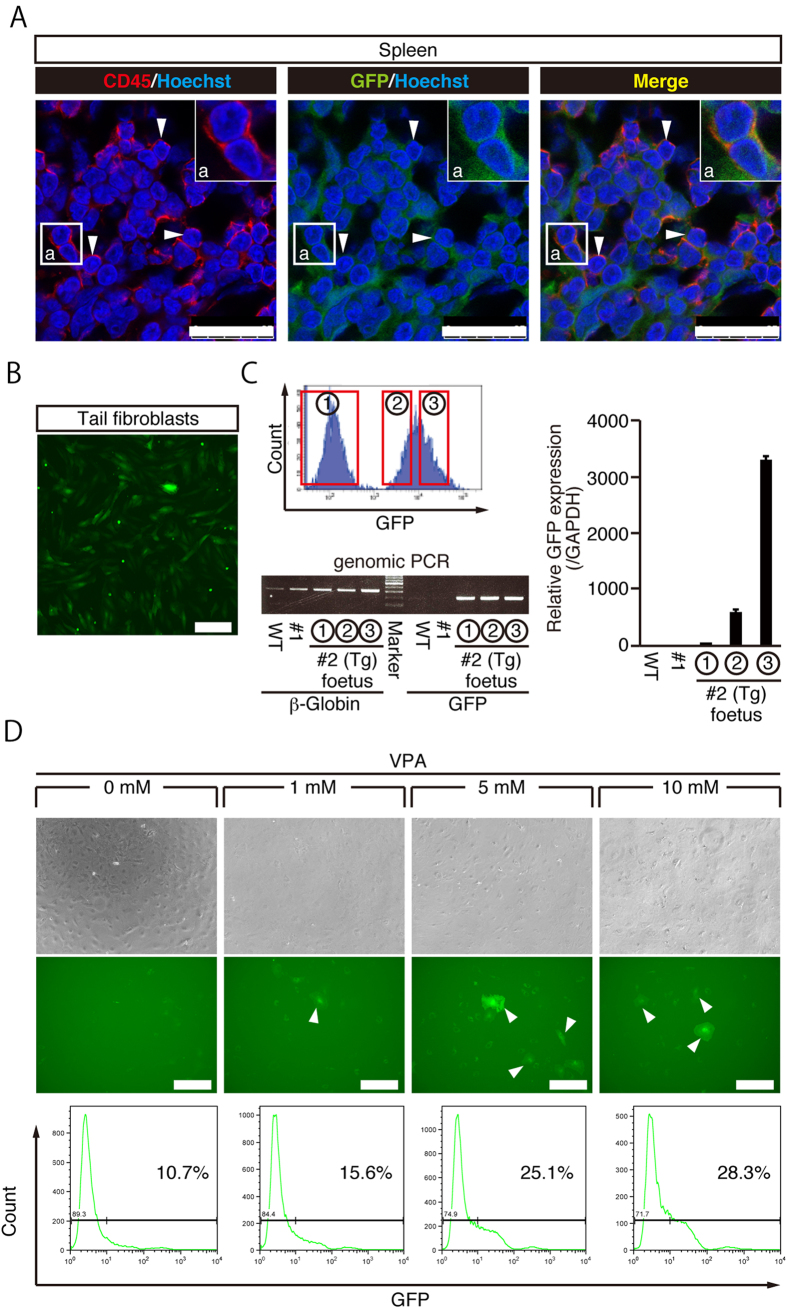
Partial GFP expression in day 92 aborted twin foetuses. (**A**) Immunohistochemistry of spleen with anti-CD45 and GFP antibodies. A subset of CD45-positive white blood cells expressed GFP protein. Colocalisation of GFP and CD45 is shown by white arrows. (Scale bar: 25 μm) (**B**). Fluorescent image of #2 tail fibroblasts. (Scale bar: 200 μm) (**C**). Sorting of #2 tail fibroblasts by GFP intensity (upper left panel). Genomic PCR results from #2-①, #2-② and #2-③ tail fibroblasts (lower left panel). GFP expression in #2 foetus fibroblasts evaluated by RT-qPCR (right panel). Data are represented as mean ± SD. (**D**). Effect of sodium valproate (VPA) on GFP expression in GFP-negative (#2-①) fibroblasts. GFP-expressing fibroblasts are indicated by white arrows. (Scale bar: 300 μm).

Previously, GFP transgenic marmoset showed partial GFP expression ranging from 0 to 19.1% in the whole peripheral blood^38^. To estimate the percentage of GFP positive cells in the peripheral blood of #2 foetus, immunohistochemistry with an anti-CD45 antibody, a marker for white blood cells, was performed on the spleen and showed that 55% of CD45 positive cells expressed GFP protein (Fig. 3A), indicating that peripheral blood cells only partially expressed GFP.

To characterize GFP positive and negative cells, we established fibroblasts from the #2 foetus tail and found that about 60% of the cells were GFP-positive by FACS analysis (Fig. 3B,C). Interestingly, we found that GFP-negative cells also carried GFP transgene, suggesting that the transgene was not transcribed (Fig. 3C). Previous studies indicated that lentiviral transgene often undergo silencing through CpG methylation, and that treatment with valproic acid, a histone deacetylase inhibitor, was effective to rescue silenced gene transcription^51^. When GFP-negative fibroblasts were cultured in the presence of valproic acid, a significant number of GFP-positive cells were observed, indicating that GFP silencing may be caused by histone deacetylation (Fig. 3D). Taken together, a GFP cynomolgus monkey foetus was successfully obtained, yet it only showed partial GFP expression and silencing of the transgene.

### Optimization of lentivirus infection on GFP expression

**Figure 4:**
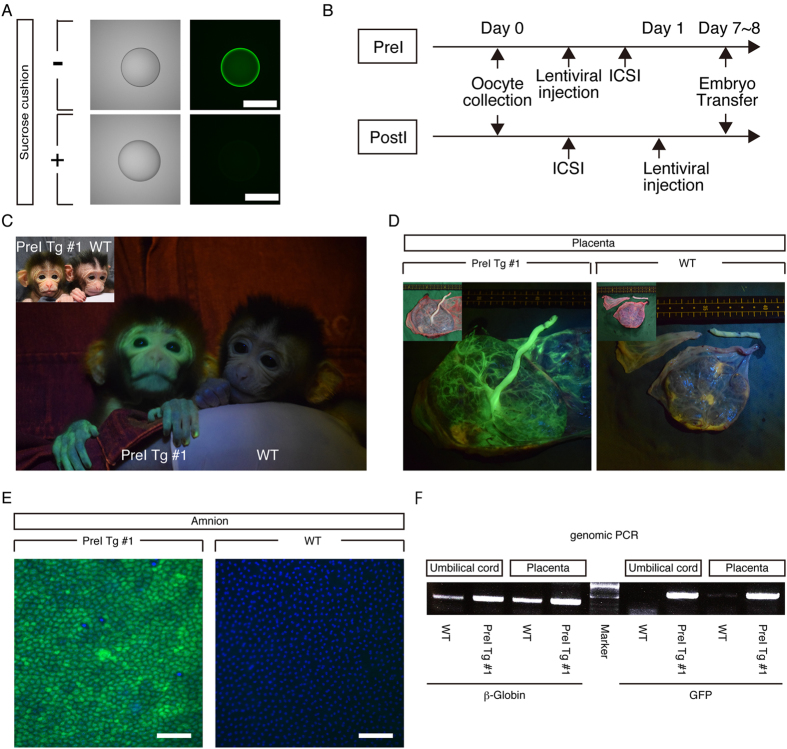
Optimization of lentivirus injection for homogenous expression of GFP in each tissue. (**A**) Fluorescent images of a virus droplet after centrifugation without (−) or with (+) a sucrose cushion. (Scale bar: 500 μm) (**B**). Optimized transgenesis protocol. PreI: Pre-ICSI. PostI: Post-ICSI. (**C**). Epifluorescence image of the face in postnatal day 2 PreI Tg #1 offspring. Inset shows a bright image of PreI Tg #1 offspring and postnatal day 1 WT offspring. (**D**) Epifluorescence images of foetus side of placenta from PreI Tg #1 offspring and a part of WT placenta. Insets show bright images. (**E**) Fluorescence images of amnion from PreI Tg #1 offspring and WT offspring. Nuclei were stained with Hoechst 33342. (Scale bar: 100 μm) (**F**). Genomic PCR analysis with DNAs of organs from PreI Tg #1 offspring and WT offspring.

To generate a transgenic cynomolgus monkey that expressed GFP homogenously in each of the various tissues, we optimized the lentivirus injection protocol. Since the lentivirus solution had a carryover of GFP protein contaminated in the process of virus production (Fig. 4A), we purified the lentivirus solution further by centrifugation in a sucrose cushion to remove GFP protein and obtained the highly purified lentiviral solution (Fig. 4A).

Wongsrikeao *et al.*, reported that when virus was injected into mature oocytes before fertilization, uniform infection was achieved in a cat embryo^46^. Therefore, matured oocytes were subjected to perivitelline-space injection with lentivirus 4 h before intracytoplasmic sperm injection (ICSI) (PreI) (Fig. 4B). After the injection, the oocytes were cultured for 4 h and subjected to ICSI and cultured until blastocyst stage (day 8).

### Full-term development of GFP cynomolgus monkeys

An offspring (named PreI Tg #1) from the PreI embryo was born successfully and showed strong fluorescence in the face and placenta compared with that of WT offspring (Fig. 4C,D). Amnion of PreI Tg #1 monkey showed homogenous GFP expression at the cellular level (Fig. 4E). PCR analysis was performed to detect GFP transgene and demonstrated that the GFP transgene was integrated in the genomic DNA from umbilical cord and placenta (Fig. 4F), demonstrating that PreI Tg #1 monkey is a GFP transgenic.

**Figure 5:**
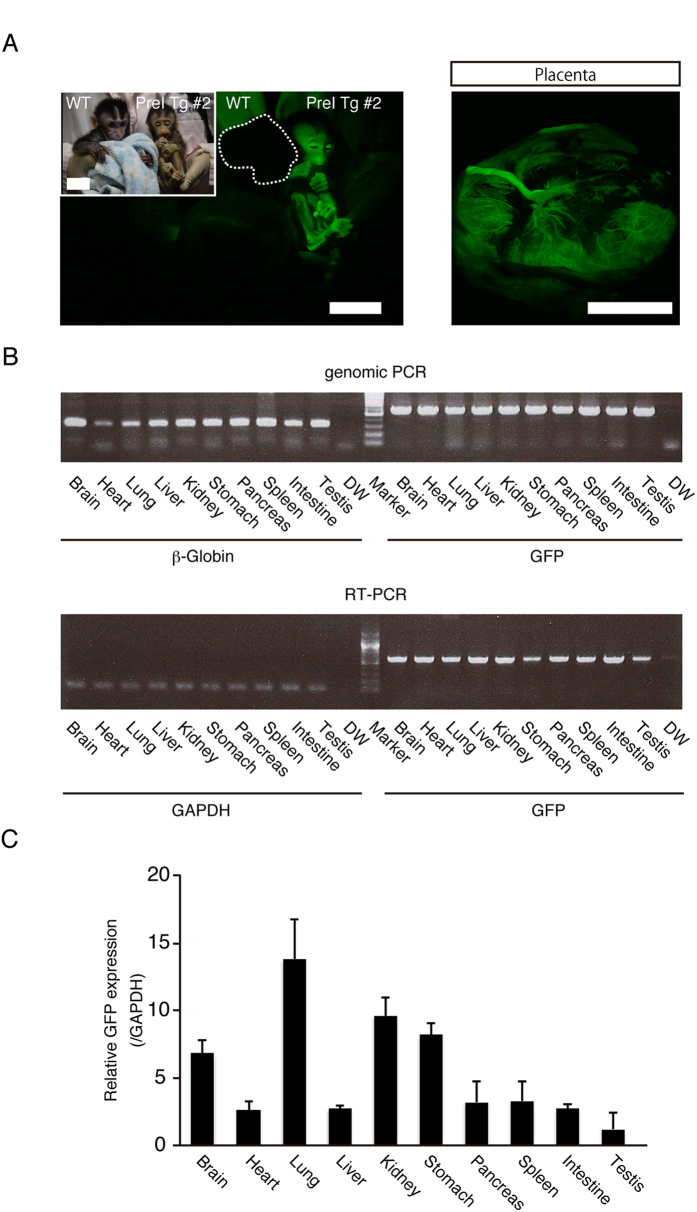
GFP expression in transgenic cynomolgus monkey offspring at postnatal day 3. (**A**) Whole body and placenta epifluorescence imaging of Pre-ICSI transgenic (PreI Tg #2) offspring. Inset shows a bright image of 3 month old WT and PreI Tg #2 offspring. (Scale bar: 5 μm) (**B**). Genomic PCR (upper panel) and RT-PCR (lower panel) results from PreI Tg #2 organs. (**C**). GFP expression of PreI Tg #2 organs evaluated by RT-qPCR. Data are represented as mean ± SD.

**Figure 6:**
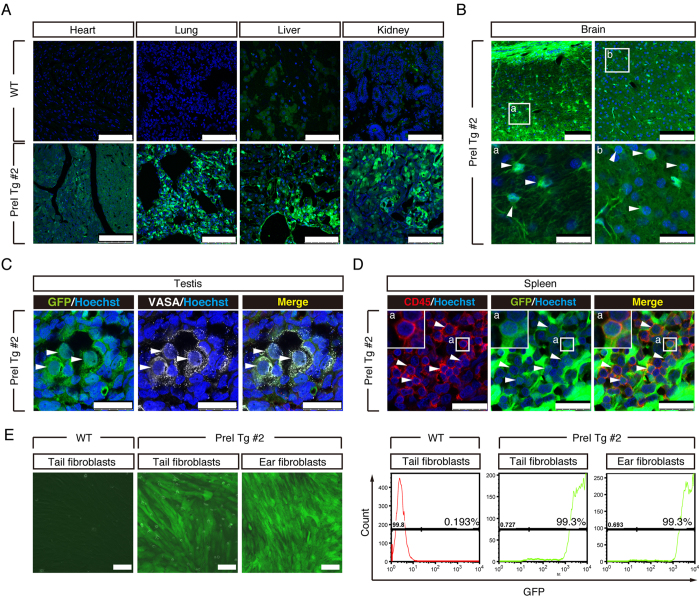
Whole body analyses of PreI Tg #2 offspring. (**A**) Expression of GFP protein revealed by immunohistochemistry with anti-GFP antibodies of PreI Tg #2 offspring by confocal microscopy. Upper and lower panel show WT and PreI Tg #2 tissues, respectively. Images were taken under the same instrumental settings (same laser intensity, etc). (Scale bar: 100 μm) (**B**). Immunohistochemical analysis of PreI Tg #2 brain with anti-GFP antibodies. Inset a: white arrows show astrocyte and GFP colocalisation. Inset b: white arrows show neuron nuclei and GFP colocalisation. (Scale bar: upper panel 100 μm, lower panel 25 μm) (**C**). Immunohistochemical staining of PreI Tg #2 testis with anti-VASA antibodies. Colocalisation of GFP and VASA is shown by white arrows. (Scale bar: upper panel 50 μm, lower panel 25 μm) (**D**). Immunohistochemistry of PreI Tg #2 spleen with anti-CD45 and GFP antibodies. Homogenous GFP protein expression in CD45-positive white blood cells was observed. Colocalisation of GFP and CD45 are shown by white arrows. (Scale bar: 25 μm) (**E**). Merged (Epifluorescence and Bright) images of fibroblasts from WT tail and PreI Tg #2 tail and ear. FACS analysis using fibroblasts from WT tail and PreI Tg #2 tail and ear. (Scale bar: 100 μm).

Although PreI Tg #1 monkey clearly showed strong GFP fluorescence in the skin and homogenous GFP expression in the amnion at the cellular level, GFP expressions in the tissues were not addressed due to consideration for animal welfare. Another offspring (named PreI Tg #2) was born, but died 3 days after birth (Table 2). Hypoplasia of the pituitary grand and pancreas were observed at the necropsy (data not shown). The whole body of the transgenic cynomolgus monkey showed strong fluorescence under an excitation light (Fig. 5A). The fluorescence was also detected in the placenta (Fig. 5A). PCR analysis indicated that GFP lentiviral transgene was integrated into the genome of various tissues (Fig. 5B). RT-PCR analysis indicated that GFP was expressed abundantly at the mRNA level in the brain, lung, kidney and stomach, while moderate GFP expression was observed in the heart, liver, pancreas, spleen, intestine and testis (Fig. 5B,C). Immunohistochemistry revealed abundant GFP expression in all tissues tested including the heart, lung, liver, kidney, spleen, stomach, and intestine (Fig. 6A, Supplementary Fig. 2A,B). This was in sharp contrast with the autofluorescence observed in WT tissues (Fig. 6A). GFP protein expressions were also evident in lateral ventricle and grey matter layer (Fig. 6B). To investigate the contribution of the transgene to the germ cell lineage, we examined GFP expression in PGCs and found that almost all VASA-positive PGCs expressed GFP (Fig. 6C). To examine the ratio of GFP positive cells in offspring, high magnification images of sections were analysed and showed that >95% of cells were GFP positive (data not shown). Furthermore, CD45 positive peripheral blood was analysed and 99% of CD45 positive cells expressed GFP (Fig. 6D). To demonstrate this more clearly, fibroblasts were established from the tail and ear of offspring, and almost all cells were GFP-positive under microscopic observation (Fig. 6E). Consistent with this, FACS analysis of fibroblasts showed that nearly 100% of cells were GFP-positive (Fig. 6E). Collectively, these results demonstrated that the GFP cynomolgus monkey created by PreI technique expressed GFP homogenously in each of the various tissues.

### Comparison of expression levels of GFP protein in tissues from Tg monkeys and the GFP mouse

**Figure 7:**
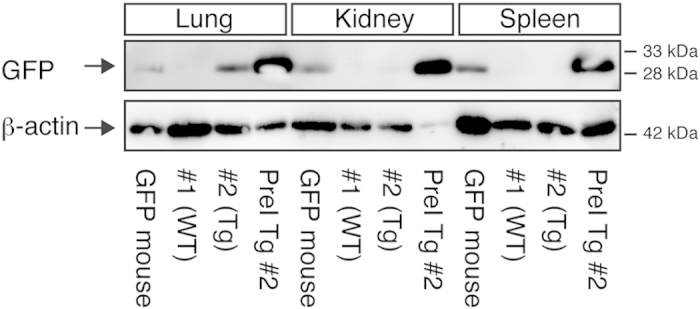
Comparison of expression levels of GFP protein from tissues in Tg monkeys and the GFP mice. Western blot was performed with 2.5 μg of proteins extracted from tissues of Tg monkeys, and the GFP mouse that carries one copy of the CAG promoter-GFP expression unit. Anti-GFP and β-actin antibodies were used to detect GFP and β-actin, respectively. Full-length blots are presented in Supplementary Fig. 3.

The green mice that ubiquitously express GFP have been created and been used for many biomedical researches such as transplantation studies^52^. Although the generation of GFP rhesus monkey and GFP marmoset have been reported^36^,^37^,^38^,^49^ previously, there was no study about the comparison of the level of GFP expression with that of the GFP mice. To evaluate the usefulness of GFP cynomolgus monkey established in this study, we compared GFP expression levels in tissues from GFP cynomolgus monkey with those from GFP mice^42^ by western blot analysis. Strong GFP expressions were detected in lung, kidney and spleen of PreI Tg cynomolgus monkey compared to those of GFP mouse (Fig. 7). #2 (Tg) foetus also expressed moderate level of GFP protein (Fig. 7). Thus, GFP cynomolgus monkeys generated in this study expressed even higher level of GFP protein in various tissues compared with those of GFP mice, and the technique established in this study will be very useful for the creation of human disease models.

## Discussion

Transgenic NHPs will be valuable for human disease models, such as Parkinson’s disease and Alzheimer diseases. So far, transgenic NHP models of Huntington’s disease^37^ and Parkinson’s disease^53^ have been created and reported. GFP is frequently used in biomedical research and GFP-expressing animals are an important source of transplantation studies. Previously, generation of GFP rhesus monkeys and GFP marmosets have been reported^36^,^37^,^38^,^49^, yet no GFP cynomolgus monkeys. To the best of our knowledge, this is the first study reporting the generation of GFP expressing cynomolgus monkeys. The strategy established in this study will be valuable for generating cynomolgus monkeys that homogenously overexpress a causative gene for human disease in each tissue.

During the generation of GFP cynomolgus monkeys, we were aware that moderate fluorescence expression is detectable even in WT tissues. It was critically important to use WT tissues as a negative control to confirm the fluorescence derived from GFP protein in transgenic tissues. It is important to note that some previous reports of GFP transgenic monkeys showed only GFP transgenic tissues but not corresponding WT tissues^36^,^37^,^49^.

Our analysis indicated that when lentivirus was injected into a two-cell-stage embryo, partial GFP expression was achieved. Although lentivirus can transduce non-dividing cells, integration is significantly slowed by the nuclear membrane^48^,^54^. This has previously been observed in other species including marmosets^38^.This mosaicism may be caused by the delay of lentiviral integration into the genome. Interestingly, a bovine study showed that the lentiviral genome could not efficiently enter the pronucleus^48^. Wongsrikeao *et al.* examined the effect of infection timing and found mosaic expression was able to be avoided by injecting lentivirus into cat oocytes before IVF^46^. Consistent with this, when lentivirus was injected into cynomolgus monkey oocytes before fertilization, rather than the two-cell embryo stage, we obtained offspring with homogenous GFP expression in each tissue, showing that lentiviral injection into mature oocytes before fertilization is an effective way to create transgenic NHPs with homogenous expression of GFP in each of the various tissues, including the amnion, and fibroblasts.

NHPs are invaluable models for high order brain research into disorders such as Parkinson’s disease, Autism and Alzheimer’s disease. Alzheimer pathology is correlated strongly with the density of activated astrocytes^55^. In these cells, the expression of GFAP is strongly upregulated^56^,^57^. In Parkinson’s disease patients, the phosphorylation level of GFAP at serine 13 was significantly lower compared with control subjects^58^. As the present study indicated that the GFP was expressed ubiquitously in various organs and cells including neuronal cells of transgenic cynomolgus monkeys, this transgenic technique will be useful to create human disease models for Parkinson’s disease and Alzheimer’s disease amongst others. The GFP cynomolgus monkey will also provide a source of green pre-implantation stage embryos, which can be used for the production of chimeric cynomolgus monkeys by the injection or aggregation with non-green embryonic stem cells.

## Materials and Methods

### Animals

Experimental procedures were approved by the Animal Care and Use Committee of Shiga University of Medical Science and methods were carried out in accordance with the approved guideline (Approval number: 26–42). Oocytes were collected from seven sexually mature female cynomolgus monkeys, aged 4–8 years and weighing 2.1–3.9 kg. Twenty sexually mature females aged 4 years old and weighing 2.0–3.8 kg, were used as recipients. Sperm were collected from one sexually mature male cynomolgus monkey, aged 12 years and weighing 6.2 kg. Temperature and humidity in the animal rooms were maintained at 25 ± 2 °C and 50 ± 5%, respectively. Monkeys were housed individually in cages (800 × 500 × 800 mm). The light cycle was 12 h of artificial light from 8 am. to 8 pm., Each animal was fed 20 g/kg of body weight of commercial pellet monkey chow (CMK-1; CLEA Japan, Inc., Tokyo, Japan) in the morning, supplemented with 20–50 g of sweet potato in the afternoon. Water was available *ad libitum*.

### Oocyte collection

Ovarian stimulation and oocyte collection were carried out as previously described by Yamasaki *et al.*^59^ with some modifications. In brief, beginning at menses, the level of sex steroid hormones was reduced by subcutaneous injection of 0.9 mg of a gonadotropin-releasing hormone antagonist (Leuplin; Takeda Chemical Industries, Ltd., Osaka, Japan). Two weeks later, Micro infusion pump (iPRECIO SMP-200, ALZET Osmotic Pumps Co., Cupertino, CA, USA) with 15 IU/kg human follicle-stimulating hormone (hFSH; Asuka Pharmaceutical Co., Tokyo, Japan) was embedded subcutaneously under anesthesia and injected 7 μl/h for 10 days. On the day after the last hFSH injection, 400 IU/kg human chorionic gonadotropin (hCG; Puberogen, Nippon Zenyaku Kogyo Co., Ltd., Fukushima, Japan) was injected intramuscularly. Oocytes were collected by follicular aspiration 40 h after hCG treatment, using a laparoscope (LA-6500, Machida Endoscope Co., Ltd., Chiba, Japan). Cumulus-oocyte complexes were recovered in Alpha modification of Eagle’s medium (MP Biomedicals LLC, Solon, OH, USA), containing 10% Serum Substitute Supplement (SSS; Irvine Scientific, Santa Ana, CA, USA) at 38 °C, in an atmosphere of humidified 5% CO_2_ and 95% in air for 1–2 h. Oocytes were stripped off cumulus cells by mechanical pipetting after brief exposure (<1 min) to 0.5 mg/mL hyaluronidase (Sigma Chemical Co., St. Louis, MO, USA), adjusted with m-TALP (pH 7.4), a modified Tyrode solution, with, lactate, pyruvate, 0.3% bovine serum albumin (Sigma Chemical Co.) and HEPES. Then, oocytes were transferred to m-TALP without hyaluronidase, at 38 °C in 5% CO_2_ until further use. Oocytes were classified by stages as germinal vesicle, metaphase I, metaphase II or degenerate.

### Intracytoplasmic sperm injection

Intracytoplasmic sperm injection was carried out on metaphase II-stage oocytes, as previously described^59^, in m-TALP containing HEPES (mTALP-HEPES). A glass needle (Humagen Fertility Diagnostics, Charlottesville, VA, USA), connected to an injector and an inverted microscope (Olympus Tokyo, Tokyo, Japan) with a micromanipulator, was used for sperm injection. Intracytoplasmic sperm injection was performed with fresh sperm collected by electric stimulation of the penis with no anaesthesia. Following ICSI, embryos were cultured in GIBCO CMRL Medium-1066 (Invitrogen, Carlsbad, CA, USA) supplemented with 20% bovine serum (Invitrogen) at 38 °C in 5% CO_2_ and 5% O_2_ then blastocyst stage embryos were generated *in vitro*.

### Lentiviral vector construction

pCSII-CAG-EGFP was constructed by introducing CAG promoter from pCAGGS and GFP cDNA into pCSII-EF-MCS-IRES2-Venus plasmid. pCAGGS was provided by Dr. Hitoshi Niwa (Kumamoto Univ.).

### Lentiviral vector package and transduction

Viral particles were obtained through Lipofectamine 2000 (Life Technologies, Calsbad, CA, USA) transfection in 293FT cells, and with packaging plasmids VSVG, RSV-Rev, HIVgp plasmids. Viral supernatants were harvested after 48 and 72 h of transfection. The supernatant then was clarified by centrifugation (1,000 × *g* for 5 min at room temperature), passed through a PVDF filter (pore size, 0.22 μm), and concentrated by ultracentrifugation (50,000 × *g* for 2 h at 4 °C). The pellet was suspended in PBS and centrifuged on a 20% (w/v) sucrose cushion. After the viral pellet was resuspended in CMRL, the infectious unit (IU) was determined by Lenti-X™ p24 Rapid Titer Kit (Takara bio, Shiga, Japan).

### Virus injection to embryos

For virus injection to the two-cell-stage embryos, two-cell-stage embryos were prepared after 24 h of ICSI and lentivirus was injected into the perivitelline space in 0.25 M sucrose/ mTALP-HEPES. For injection to oocytes, metaphase II stage oocytes were selected and lentivirus was injected into the perivitelline space in 0.25 M sucrose/ mTALP-HEPES. After 4 h of virus injection, ICSI was performed.

### Embryo transfer and pregnancy detection

When embryos developed to expanded blastocysts, one or two embryos were transferred into each female recipient. Embryo transfer was performed as previously described^59^, with some modifications. Laparoscopy was carried out on alternate days from days 10–14 of the menstrual cycle after menstruation (ovulation day = day 0) until ovulation occurred. Recipients were selected for embryo transfer 1–6 day after ovulation. Neither the stage of the embryos nor the number of days after the recipient’s ovulation were matched. Embryos were aspirated into a catheter (ETC3040SM5-17; Kitazato Medical Service Co., Ltd., Tokyo, Japan) under a stereomicroscope. The catheter was inserted into the oviduct of the recipient via the fimbria under the laparoscope, and the cultured embryo was transplanted with a small amount of medium. Pregnancy was determined by ultrasonography 30 days after ICSI.

### RT- quantitative PCR

Total RNA was extracted from cells or tissues using RNeasy Mini kits (Qiagen, Hilden, Germany). For reverse transcription, ReverTra Ace (Toyobo Co., Ltd, Osaka, Japan) and oligo (dT) 20 primer were used. For real-time PCR, THUNDERBIRD SYBR qPCR Mix (Toyobo) was used. Transcript levels were determined in triplicate reactions and normalized against the corresponding levels of GAPDH. Primer sequences are shown in Supplementary Table 1.

### Immunohistochemical analysis

Tissues were fixed by 4% PFA at 4 °C overnight, embedded in OCT compound, frozen in liquid nitrogen, sliced into 10-μm sections and placed onto glass slides that were treated with Blocking One for 30 min at room temperature. Primary antibodies and dilutions used were rabbit anti-GFP AlexaFluor 488 conjugate (1:400, Life Technologies A21311), mouse anti-Oct4 (1:400, Santa Cruz sc-5279), mouse anti-NeuN (1:500, Millipore MAB377), rabbit anti-GFAP (1:500, Biomedical Technologies Inc. BT-575), rabbit anti-CD45 (1:100, Dako M0701), rabbit anti-DDX4 (VASA) (1:400, Abcam ab13840), which were then detected with appropriate secondary AlexaFluor 488, 568 or 647 antibodies. Cells were counterstained with Hoechst 33342 and observed using a Leica TCS SP8 confocal microscope.

### FACS analysis

Tail fibroblasts were established from day 92 foetuses (#1 and #2) and day 3 offspring (PreI Tg #2). After removal of tail skin, the remaining tails were washed in PBS and maintained in Dulbecco’s modified eagle medium (DMEM, Invitrogen) containing 10% foetal bovine serum (FBS, JRH Biosciences, Corston Bath, UK), penicillin, streptomycin (Invitrogen), and primocin (Invivogen). Ear fibroblasts were established from PreI Tg #2 by cutting ears into 5 mm^3^ fragments, washing in PBS and then cultured in DMEM containing 10% FBS, penicillin, streptomycin, and primocin. The fibroblasts were cultured until confluent and then collected by trypsin treatment. Cells were resuspended in a final volume of 500 μl of PBS/2% FCS and analysed with a Becton Dickinson FACSAria cell sorter.

### Observation of green fluorescence in transgenic offspring

Images were captured under 470 nm excitation light with 520-nm wavelength filters using a Nikon D3300 digital camera. No image intensifying procedures were applied to any of the images. A 1-day-old and 3-month-old cynomolgus monkeys were used as a negative control for PreI Tg #1 and #2 respectively.

### Western blot analysis

One mm^2^ of tissues were incubated in RIPA buffer (50 mM Tris-HCl, 150 mM NaCl, 0.5% sodium deoxycholate, 1% NP40 and 0.1% SDS) with protease and phosphatase inhibitors for 10 min at 4 °C. After centrifugation (15,000 × *g* for 5 min), protein samples were obtained from supernatant. Samples were diluted in sample buffer (Wako, Osaka, Japan) at a final concentration (10 μg/μl) and stored at −20 °C just before assessment. After denaturing by boiling at 95 °C for 5 min, 2.5 μl of sample was separated by SDS-PAGE on 10% polyacrylamide gel at 250 V for 80 min and then transferred onto a polyvinylidene fluoride membrane (Merck Millipore, Darmstadt, Germany). The membrane was blocked using Blocking One, and then incubated with rabbit anti-GFP AlexaFluor 488 conjugate (1:2,000) or rabbit anti-β-actin horseradish peroxidase (HRP) conjugate (1:4,000) antibody overnight at 4 °C in Blocking One. After three washes in Tween20-PBS (T-PBS), the membrane incubated with rabbit anti-GFP was treated with HRP-labeled anti-rabbit immunoglobulin G (1:2,000; Invitrogen) in Blocking One for 1 h at room temperature. After one wash of 15 min and five washes of 5 min each with T-PBS, peroxidase activity was visualized using the Chemi-Lumi One Super (Nacalai Tesque) according to the manufacturer’s instructions. Tissues from a GFP mouse (C57BL/6-Tg(CAG-EGFP)10sb/J)^42^ that carries one copy of the CAG promoter-GFP expression unit were provided by Drs. H. Kojima and M. Katagi (Shiga University of Medical Science) and used as a positive control.

### Statistical analysis

Statistical analyses of all data comparisons were carried out using the t-test using Excel software. *P* < 0.05 was considered statistically significant.

## Additional Information

**How to cite this article**: Seita, Y. *et al.* Generation of transgenic cynomolgus monkeys that express green fluorescent protein throughout the whole body. *Sci. Rep.* **6**, 24868; doi: 10.1038/srep24868 (2016).

## Supplementary Material

Supplementary Information

## Figures and Tables

**Table 1:** *In vitro* and *in vivo* development of cynomolgus monkey embryo after lentivirus injection.

Injection timing of virus	24 h post ICSI
No. of embryos used	18
No. of 2 cell stage	18
No. of blastocyst (% per 2 cell)	5 (27.8)
ETs	5
No. of surrogates	3
No. of pregnancies at 30 days after ICSI	2
Multiple pregnancy	1 (twin)
Aborted	2*
Live birth	0

ET: embryo transfer.

^*^One of the aborted foetus at 35 days after ICSI that was only placenta and the other foetus at 92 days after ICSI that was twin.

**Table 2:** *In vitro* and *in vivo* development of PreI embryo.

Group	PreI	Control
Injection timing of virus	4 h pre ICSI	–
No. of embryos used	69	34
No. of 2 cell stage	56	18
No. of blastocyst (% per 2 cell)	25 (44.6)	10 (55.6)
ETs	17	–
No. of surrogates	17	–
No. of pregnancies at 30 days after ICSI	5	–
Spontaneous miscarriage	3	–
Live birth	2*	–

PreI: Pre-ICSI.

ET: embryo transfer.

^*^One offspring died three days after birth.

## References

[b1] Jaenisch R. & Mintz B. Simian virus 40 DNA sequences in DNA of healthy adult mice derived from preimplantation blastocysts injected with viral DNA. Proc. Natl. Acad. Sci. USA 71, 1250–1254 (1974). 4364530 10.1073/pnas.71.4.1250 PMC388203

[b2] Gordon J. W., Scangos G. A., Plotkin D. J. & Barbosa J. A. & Ruddle, F. H. Genetic transformation of mouse embryos by microinjection of purified DNA. Proc. Natl. Acad. Sci. USA 77, 7380–7384 (1980). 6261253 10.1073/pnas.77.12.7380 PMC350507

[b3] Bachmann S., Peters J., Engler E., Ganten D. & Mullins J. Transgenic rats carrying the mouse renin gene–morphological characterization of a low-renin hypertension model. Kidney Int. 41, 24–36 (1992). 1593860 10.1038/ki.1992.4

[b4] Mullins J. J., Peters J. & Ganten D. Fulminant hypertension in transgenic rats harbouring the mouse Ren-2 gene. Nature 344, 541–544 (1990). 2181319 10.1038/344541a0

[b5] Hammer R. E. *et al.* Production of transgenic rabbits, sheep and pigs by microinjection. Nature 315, 680–683 (1985). 3892305 10.1038/315680a0

[b6] Brinster R. L. *et al.* Somatic expression of herpes thymidine kinase in mice following injection of a fusion gene into eggs. Cell 27, 223–231 (1981). 6276022 10.1016/0092-8674(81)90376-7 PMC4883678

[b7] Wagner T. E. *et al.* Microinjection of a rabbit beta-globin gene into zygotes and its subsequent expression in adult mice and their offspring. Proc. Natl. Acad. Sci. USA 78, 6376–6380 (1981). 6796959 10.1073/pnas.78.10.6376 PMC349042

[b8] Doetschman T. *et al.* Targetted correction of a mutant HPRT gene in mouse embryonic stem cells. Nature 330, 576–578 (1987). 3683574 10.1038/330576a0

[b9] Kuehn M. R., Bradley A., Robertson E. J. & Evans M. J. A potential animal model for Lesch-Nyhan syndrome through introduction of HPRT mutations into mice. Nature 326, 295–298 (1987). 3029599 10.1038/326295a0

[b10] Springer M. S., Murphy W. J., Eizirik E. & O’Brien S. J. Placental mammal diversification and the Cretaceous-Tertiary boundary. Proc. Natl. Acad. Sci. USA 100, 1056–1061 (2003). 12552136 10.1073/pnas.0334222100 PMC298725

[b11] Kalter S. S. & Heberling R. L. Serologic response of primates to influenza viruses. Proc. Soc. Exp. Biol. Med. 159, 414–417 (1978). 733806 10.3181/00379727-159-40360

[b12] Bouvier N. M. & Lowen A. C. Animal Models for Influenza Virus Pathogenesis and Transmission. Viruses 2, 1530–1563 (2010). 21442033 10.3390/v20801530 PMC3063653

[b13] Tripp R. A. & Tompkins S. M. Animal models for evaluation of influenza vaccines. Curr. Top. Microbiol. Immunol. 333, 397–412 (2009). 19768416 10.1007/978-3-540-92165-3_19

[b14] Fukushi M. *et al.* Serial histopathological examination of the lungs of mice infected with influenza A virus PR8 strain. PLos One 6, e21207 (2011). 21701593 10.1371/journal.pone.0021207 PMC3118813

[b15] Seok J. *et al.* Genomic responses in mouse models poorly mimic human inflammatory diseases. Proc. Natl. Acad. Sci. USA 110, 3507–3512 (2013). 23401516 10.1073/pnas.1222878110 PMC3587220

[b16] Wenzel S. & Holgate S. T. The mouse trap: It still yields few answers in asthma. American Journal of Respiratory and Critical Care Medicine 174, 1173–1176 (2006). 17110654 10.1164/rccm.2609002

[b17] Gautier C. A., Kitada T. & Shen J. Loss of PINK1 causes mitochondrial functional defects and increased sensitivity to oxidative stress. Proc. Natl. Acad. Sci. USA 105, 11364–11369 (2008). 18687901 10.1073/pnas.0802076105 PMC2516271

[b18] Goldberg M. S. *et al.* Parkin-deficient mice exhibit nigrostriatal deficits but not loss of dopaminergic neurons. J. Biol. Chem. 278, 43628–43635 (2003). 12930822 10.1074/jbc.M308947200

[b19] Andres-Mateos E. *et al.* DJ-1 gene deletion reveals that DJ-1 is an atypical peroxiredoxin-like peroxidase. Proc. Natl. Acad. Sci. USA 104, 14807–14812 (2007). 17766438 10.1073/pnas.0703219104 PMC1976193

[b20] Hinkle K. M. *et al.* LRRK2 knockout mice have an intact dopaminergic system but display alterations in exploratory and motor co-ordination behaviors. Mol. Neurodegener. 7, 25 (2012). 22647713 10.1186/1750-1326-7-25 PMC3441373

[b21] Hennis M. R., Marvin M. A., Taylor C. M. & Goldberg M. S. Surprising behavioral and neurochemical enhancements in mice with combined mutations linked to Parkinson’s disease. Neurobiol. Dis. 62, 113–123 (2014). 24075852 10.1016/j.nbd.2013.09.009 PMC3900415

[b22] Rangarajan A. & Weinberg R. A. Opinion: Comparative biology of mouse versus human cells: modelling human cancer in mice. Nat. Rev. Cancer 3, 952–959 (2003). 14737125 10.1038/nrc1235

[b23] Abbott D. H. & Hearn J. P. Physical, hormonal and behavioural aspects of sexual development in the marmoset monkey, Callithrix jacchus. J. Reprod. Fertil. 53, 155–166 (1978). 417178 10.1530/jrf.0.0530155

[b24] Gromoll J. *et al.* A new subclass of the luteinizing hormone/chorionic gonadotropin receptor lacking exon 10 messenger RNA in the New World monkey (Platyrrhini) lineage. Biol. Reprod. 69, 75–80 (2003). 12606382 10.1095/biolreprod.102.014902

[b25] Binkley N. *et al.* Zoledronate prevents the development of absolute osteopenia following ovariectomy in adult rhesus monkeys. J. Bone Miner. Res. 13, 1775–1782 (1998). 9797488 10.1359/jbmr.1998.13.11.1775

[b26] Tardif S. D. & Jaquish C. E. Number of ovulations in the marmoset monkey (Callithrix jacchus): relation to body weight, age and repeatability. Am. J. Primatol. 42, 323–329 (1997). 9261513 10.1002/(SICI)1098-2345(1997)42:4<323::AID-AJP7>3.0.CO;2-Z

[b27] Tardif S. D. *et al.* Characterization of obese phenotypes in a small nonhuman primate, the common marmoset (Callithrix jacchus). Obesity (Silver Spring). 17, 1499–1505 (2009). 19325546 10.1038/oby.2009.77 PMC3823549

[b28] Monsein L. H. *et al.* Irreversible regional cerebral ischemia: serial MR imaging and proton MR spectroscopy in a nonhuman primate model. Ajnr Am. J. Neuroradiol. 14, 963–970 (1993). 8352171 PMC8333825

[b29] Marshall J. W. B. *et al.* Serial MRI, functional recovery, and long-term infarct maturation in a non-human primate model of stroke. Brain Res. Bull. 61, 577–585 (2003). 14519454 10.1016/s0361-9230(03)00214-4

[b30] Barraud Q. *et al.* Sleep disorders in Parkinson’s disease: the contribution of the MPTP non-human primate model. Exp. Neurol. 219, 574–582 (2009). 19635479 10.1016/j.expneurol.2009.07.019

[b31] Soderstrom K., O’Malley J., Steece-Collier K. & Kordower J. H. Neural repair strategies for Parkinson’s disease: insights from primate models. Cell Transplant. 15, 251–265 (2006). 16719060 10.3727/000000006783982025

[b32] DeLong M. R. Primate models of movement disorders of basal ganglia origin. Trends Neurosci. 13, 281–285 (1990). 1695404 10.1016/0166-2236(90)90110-v

[b33] Isacson O., Riche D., Hantraye P., Sofroniew M. V. & Maziere M. A primate model of Huntington’s disease: cross-species implantation of striatal precursor cells to the excitotoxically lesioned baboon caudate-putamen. Exp. brain Res. 75, 213–220 (1989). 2523313 10.1007/BF00248544

[b34] Kisu I. *et al.* Uterus allotransplantation in cynomolgus macaque: a preliminary experience with non-human primate models. J. Obstet. Gynaecol. Res. 40, 907–918 (2014). 24612366 10.1111/jog.12302

[b35] Enskog A. *et al.* Uterus transplantation in the baboon: methodology and long-term function after auto-transplantation. Hum. Reprod. 25, 1980–1987 (2010). 20519250 10.1093/humrep/deq109

[b36] Chan A. W., Chong K. Y., Martinovich C., Simerly C. & Schatten G. Transgenic monkeys produced by retroviral gene transfer into mature oocytes. Science 291, 309–312 (2001). 11209082 10.1126/science.291.5502.309

[b37] Yang S.-H. *et al.* Towards a transgenic model of Huntington’s disease in a non-human primate. Nature 453, 921–924 (2008). 18488016 10.1038/nature06975 PMC2652570

[b38] Sasaki E. *et al.* Generation of transgenic non-human primates with germline transmission. Nature 459, 523–527 (2009). 19478777 10.1038/nature08090

[b39] Niu Y. *et al.* Generation of gene-modified cynomolgus monkey via Cas9/RNA-mediated gene targeting in one-cell embryos. Cell 156, 836–843 (2014). 24486104 10.1016/j.cell.2014.01.027

[b40] Liu H. *et al.* TALEN-mediated gene mutagenesis in rhesus and cynomolgus monkeys. Cell Stem Cell 14, 323–328 (2014). 24529597 10.1016/j.stem.2014.01.018 PMC4024384

[b41] Chen Y. *et al.* Functional disruption of the dystrophin gene in rhesus monkey using CRISPR/Cas9. Hum. Mol. Genet. 24, 3764–3774 (2015). 25859012 10.1093/hmg/ddv120 PMC5007610

[b42] Okabe M., Ikawa M., Kominami K., Nakanishi T. & Nishimune Y. ‘Green mice’ as a source of ubiquitous green cells. FEBS Lett. 407, 313–319 (1997). 9175875 10.1016/s0014-5793(97)00313-x

[b43] Hakamata Y. *et al.* Green fluorescent protein-transgenic rat: a tool for organ transplantation research. Biochem. Biophys. Res. Commun. 286, 779–785 (2001). 11520065 10.1006/bbrc.2001.5452

[b44] Wang H. J., Lin A. X., Zhang Z. C. & Chen Y. F. Expression of porcine growth hormone gene in transgenic rabbits as reported by green fluorescent protein. Anim. Biotechnol. 12, 101–110 (2001). 11808625 10.1081/ABIO-100108336

[b45] Takahashi R. *et al.* Establishment and characterization of CAG/EGFP transgenic rabbit line. Transgenic Res. 16, 115–120 (2007). 17103241 10.1007/s11248-006-9043-1

[b46] Wongsrikeao P., Saenz D., Rinkoski T., Otoi T. & Poeschla E. Antiviral restriction factor transgenesis in the domestic cat. Nat. Methods 8, 853–859 (2011). 21909101 10.1038/nmeth.1703 PMC4006694

[b47] Naruse K. *et al.* Production of a transgenic pig expressing human albumin and enhanced green fluorescent protein. J. Reprod. Dev. 51, 539–546 (2005). 15947455 10.1262/jrd.16073

[b48] Reichenbach M. *et al.* Germ-line transmission of lentiviral PGK-EGFP integrants in transgenic cattle: new perspectives for experimental embryology. Transgenic Res. 19, 549–556 (2010). 19862638 10.1007/s11248-009-9333-5

[b49] Niu Y. *et al.* Transgenic rhesus monkeys produced by gene transfer into early-cleavage-stage embryos using a simian immunodeficiency virus-based vector. Proc. Natl. Acad. Sci. USA 107, 17663–17667 (2010). 20870965 10.1073/pnas.1006563107 PMC2955145

[b50] Niwa H., Yamamura K. & Miyazaki J. Efficient selection for high-expression transfectants with a novel eukaryotic vector. Gene 108, 193–199 (1991). 1660837 10.1016/0378-1119(91)90434-d

[b51] Pikaart M. J. & Felsenfeld G. Loss of transcriptional activity of a transgene is accompanied by DNA methylation and histone deacetylation and is prevented by insulators. Genes & Dev. 12, 2852–2862 (1998). 9744862 10.1101/gad.12.18.2852 PMC317165

[b52] Herbst F. *et al.* Extensive methylation of promoter sequences silences lentiviral transgene expression during stem cell differentiation *in vivo*. Mol. Ther. 20, 1014–1021 (2012). 22434137 10.1038/mt.2012.46 PMC3345972

[b53] Niu Y. *et al.* Early Parkinson ’ s disease symptoms in α -synuclein transgenic monkeys. Hum. Mol. Genet. 24, 2308–2317 (2015). 25552648 10.1093/hmg/ddu748 PMC4375423

[b54] Naldini L. *et al.* *In vivo* gene delivery and stable transduction of nondividing cells by a lentiviral vector. Science 272, 263–267 (1996). 8602510 10.1126/science.272.5259.263

[b55] Muramori F., Kobayashi K. & Nakamura I. A quantitative study of neurofibrillary tangles, senile plaques and astrocytes in the hippocampal subdivisions and entorhinal cortex in Alzheimer’s disease, normal controls and non-Alzheimer neuropsychiatric diseases. Psychiatry Clin. Neurosci. 52, 593–599 (1998). 9895207 10.1111/j.1440-1819.1998.tb02706.x

[b56] Hanzel D. K., Trojanowski J. Q., Johnston R. F. & Loring J. F. High-throughput quantitative histological analysis of Alzheimer’s disease pathology using a confocal digital microscanner. Nat. Biotechnol. 17, 53–57 (1999). 9920269 10.1038/5225

[b57] Beach T. G., Walker R. & McGeer E. G. Patterns of gliosis in Alzheimer’s disease and aging cerebrum. Glia 2, 420–436 (1989). 2531723 10.1002/glia.440020605

[b58] Clairembault T. *et al.* Enteric GFAP expression and phosphorylation in Parkinson’s disease. Journal of Neurochemistry 130, 805–815 (2014). 24749759 10.1111/jnc.12742

[b59] Yamasaki J. *et al.* Vitrification and transfer of cynomolgus monkey (Macaca fascicularis) embryos fertilized by intracytoplasmic sperm injection. Theriogenology 76, 33–38 (2011). 21529920 10.1016/j.theriogenology.2011.01.010

