# Supplementary material for: Generation of transgenic cynomolgus monkeys that express green fluorescent protein throughout the whole body
Source: Sci Rep. 2016 Apr 25;6:24868. doi: 10.1038/srep24868 (PMC4843004; doi:10.1038/srep24868)
Supplement: Supplementary Information [file srep24868-s1.pdf]

Supplementary Information

Generation of transgenic cynomolgus monkeys that express green fluorescent protein throughout the whole body

Yasunari Seita[1, 2], Tomoyuki Tsukiyama[1], Chizuru Iwatani[1, 2], Hideaki Tsuchiya[1], Jun Matsushita[1, 2], Takuya Azami[3], Junko Okahara[4], Shinichiro Nakamura[1], Yoshitaka Hayashi[5], Seiji Hitoshi[5], Yasushi Itoh[6], Takeshi Imamura[7], Masaki Nishimura[8], Ikuo Tooyama[8], Hiroyuki Miyoshi[9], Mitinori Saitou[2, 10, 11, 12], Kazumasa Ogasawara[6], Erika Sasaki[4], and Masatsugu Ema[1, 13*]

Figure Legends

Supplementary Figure 1. Autofluorescence in wild type monkey tissues

A. Immunohistochemistry of tissues from a day 92 aborted twin foetus detected by confocal microscopy. (Scale bar: 250 μm) B. Effect of laser intensity on the fluorescence tissue images detected by confocal microscopy. Inset a, b and c images show renal tubules in the kidney. Inset d and e images show hepatocytes in the liver. Inset f images show the central vein in the liver. (Scale bar: 250 μm). C. WT monkey autofluoresces as if it is a GFP Tg monkey. Epifluorescence images of a WT monkey were taken at different exposure time under excitation light (489 nm)

Supplementary Figure 2. GFP protein expression in various tissues of day 3 PreI Tg #2 offspring

A. Immunohistochemistry of tissues from a day 3 PreI Tg #2 offspring detected by confocal microscopy. N. A.; Not Analysed. (Scale bar: 100 μm) B. Immunohistochemistry of tissues from a day 3 PreI Tg #2 offspring detected by confocal microscopy with high magnification. (Scale bar: 25 μm)

Supplementary Figure 3. Comparison of expression levels of GFP protein from tissues in Tg monkeys and the GFP mice

Whole blot of fig. 7 pictures. GFP and β-actin bands were cropped from these whole blots. Cropped lines were shown with boxes.

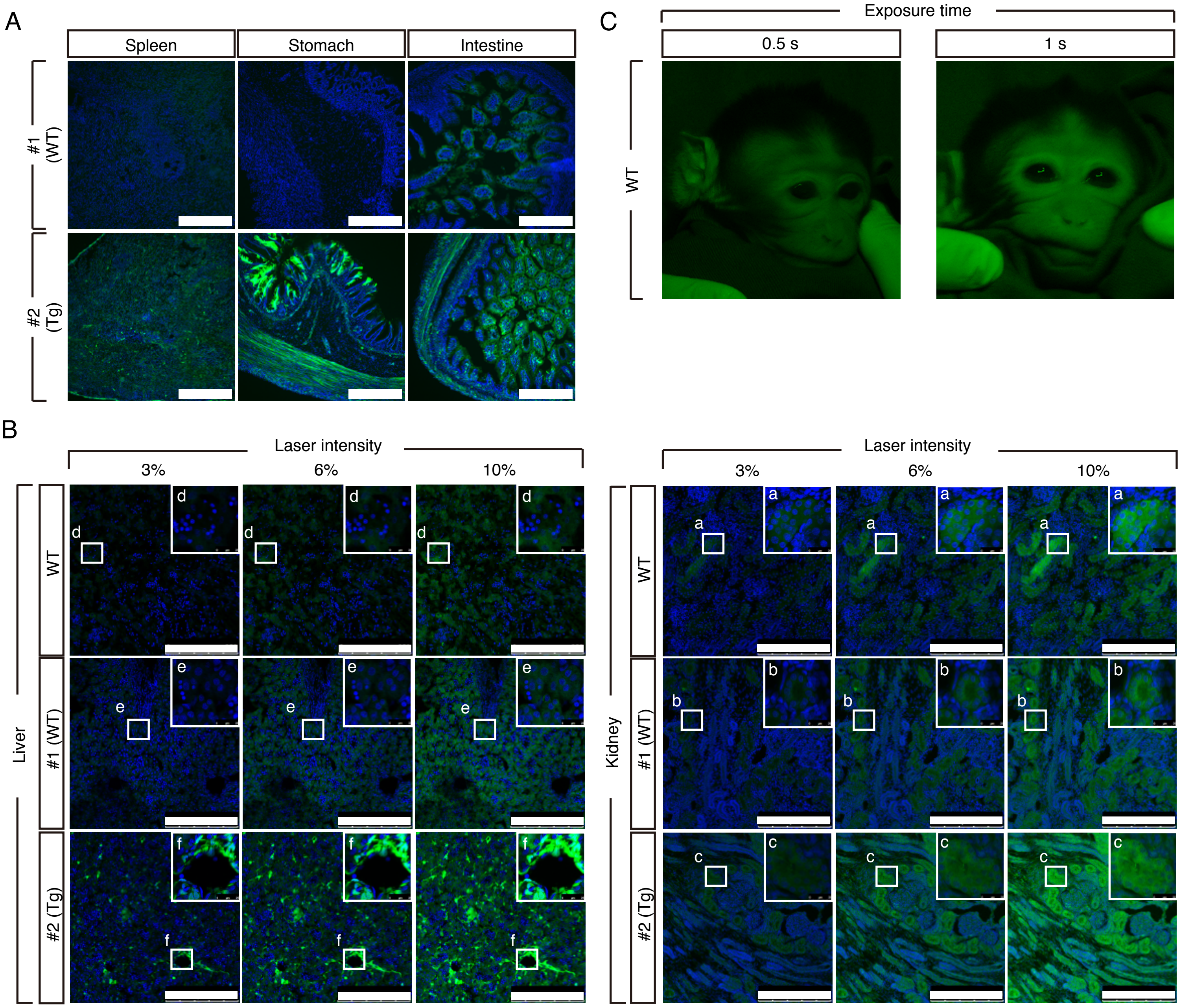

Seita *et al*., Supplementary Fig. 1

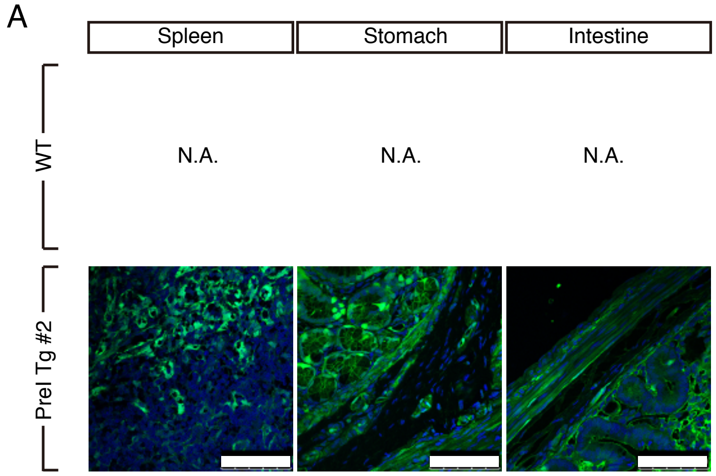

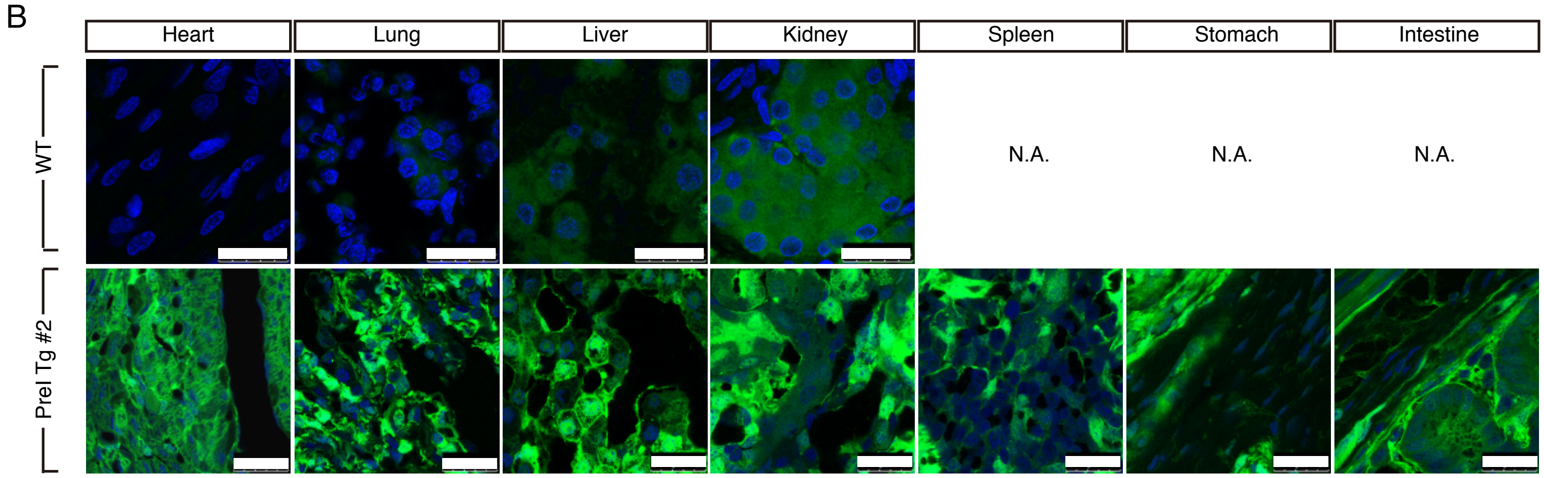

Seita *et al*., Supplementary Fig. 2

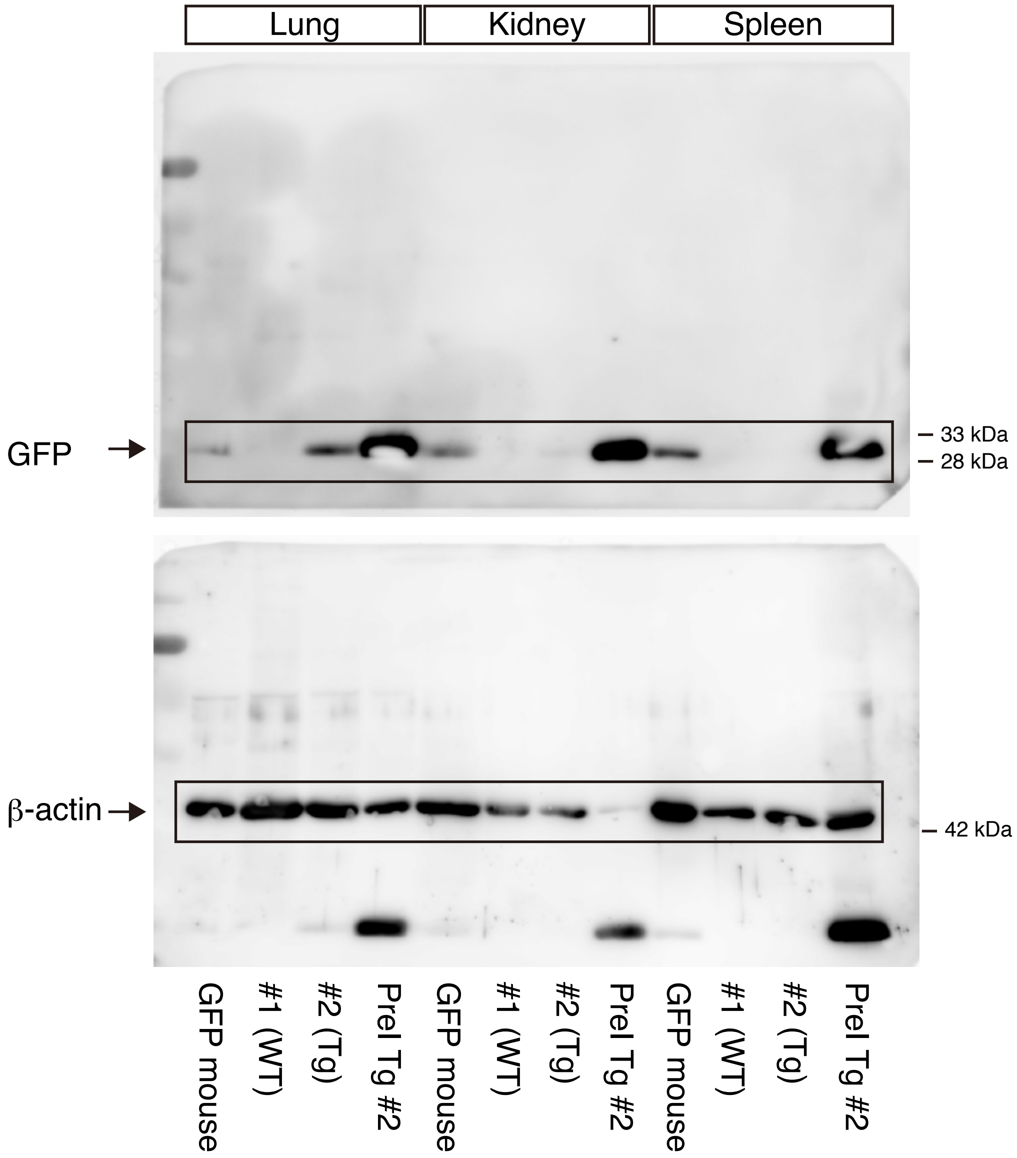

Seita *et al.*, Supplementary Fig. 3

Table S1: List of PCR primer sequences

Gene	Primer Sequence (Forward)	Primer Sequence (Reverse)
GFP	GCGACGTAAACGGCCACAAGTTCAGC	CTGGGTGCTCAGGTAGTGGTTGTCG
β-globin	GATGAAGTTGGTGAGGC	ACCCTTGAGGTTGTCCAGGT
GAPDH	TTCAACAGCGACACCCACTC	GTTGCTGTAGCCAAATTCGTTG
